# 1585. The Annual Cost per Newly Virally-Suppressed Person with HIV in Those Starting Long-Acting Injectable Antiretroviral Therapy with Detectable Viremia in a Safety-Net Clinic in San Francisco

**DOI:** 10.1093/ofid/ofad500.1420

**Published:** 2023-11-27

**Authors:** Elliot Marseille, Ryan Walker, Jon Oskarsson, Janet Grochowski, Matt Hickey, Elizabeth V Imbert, John Szumowski, Kimberly Koester, Mollie Smith, Tor Neilands, Mallory Johnson, John Sauceda, Elizabeth Montgomery, Moira McNulty, Jonathan Colasanti, Katerina Christopoulos

**Affiliations:** University of California, Berkeley, Berkeley, California; University of California, San Francisco, San Francisco, California; University of California, San Francisco, San Francisco, California; University of California, San Francisco, San Francisco, California; University of California, San Francisco, San Francisco, California; UCSF, San Francisco, CA; University of California, San Francisco, San Francisco, California; University of California, San Francisco, San Francisco, California; University of California, San Francisco, San Francisco, California; University of California, San Francisco, San Francisco, California; University of California, San Francisco, San Francisco, California; University of California San Francisco, San Francisco, California; University of California, San Francisco, San Francisco, California; University of Chicago, Chicago, Illinois; EMORY UNIVERSITY SCHOOL OF MEDICINE, Atlanta, Georgia; University of California San Francisco, San Francisco, California

## Abstract

**Background:**

Long-acting injectable cabotegravir/rilpivirine (CAB/RPV-LA) holds promise to effectively treat people with HIV (PWH) who are unable to achieve or maintain viral suppression on oral antiretroviral therapy (ART).

**Methods:**

We employed standard micro-costing techniques to identify the resources and associated annual costs to provide CAB/RPV-LA to those initiating it with detectable viremia at San Francisco General Hospital’s Ward 86 clinic, accounting for q4week vs. q8week injections. Through structured interviews with service providers and administrators, we elicited the time in minutes for each staff member to perform each task, using mid-points for ranges. Person-minutes were applied to the personnel compensation rate to derive the total personnel cost per person. One-time costs required for CAB/RPV-LA initiation were distributed over 29.3 years, the expected average duration of treatment. We used the Medi-Cal reimbursement rate for the cost of CAB/RPV-LA and a literature-based estimate of other medical care costs of treating people with viral suppression to derive the total variable cost of CAB/RPV-LA treatment from the payer’s perspective. This result was adjusted by the viral suppression success rate at Ward 86 (96.5%) to derive the cost per new virally-suppressed PWH. We deducted the cost of standard ART and medical care to derive the incremental cost per additional virally suppressed PWH. Costs were adjusted to 2022 dollars.

**Results:**

The annual variable cost of providing CAB/RPV-LA was $36,511, $35,252 for CAB/RPV-LA medication and $1,259 for other variable costs, mainly personnel. Including other medical care costs yielded a total annual medical care cost of $43,945 per newly virally-suppressed person. Deducting the cost of standard care, $42,276, from this sum suggests an incremental annual variable cost for CAB/RPV-LA of $1,668 per newly virally-suppressed person. If the cost of CAB/RPV-LA were reduced slightly, e.g. to 90% of current cost, CAB/RPV-LA would generate net savings of $1,985.

Table 1

**Figure:**
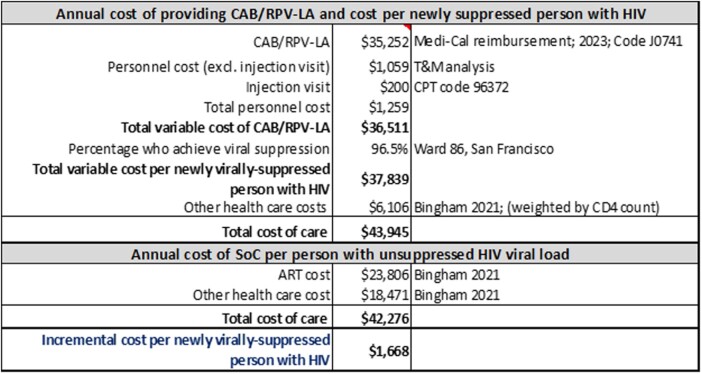

Annual Cost of Providing CAB/RPV-LA and Cost per Newly Suppressed Person with HIV

**Conclusion:**

We found that the additional cost per newly-suppressed PWH was $1,668. CAB/RPV is likely to be highly cost-effective for this population. Modest reductions in CAB/RPV-LA price could render it cost-saving from a health care payer perspective.

**Disclosures:**

**Jonathan Colasanti, MD, MSPH**, DKB MED LLC: Honoraria|Prime Education LLC: Advisor/Consultant **Katerina Christopoulos, MD, MPH**, Gilead Sciences: Advisor/Consultant

